# Single Nucleotide Polymorphism Genotyping in Single‐Molecule Electronic Circuits

**DOI:** 10.1002/advs.201700158

**Published:** 2017-07-26

**Authors:** Gen He, Jie Li, Chuanmin Qi, Xuefeng Guo

**Affiliations:** ^1^ Beijing National Laboratory for Molecular Sciences State Key Laboratory for Structural Chemistry of Unstable and Stable Species College of Chemistry and Molecular Engineering Peking University Beijing 100871 P. R. China; ^2^ Key Laboratory of Radiopharmaceuticals Ministry of Education College of Chemistry Beijing Normal University Beijing 100875 P. R. China; ^3^ Department of Materials Science and Engineering College of Engineering Peking University Beijing 100871 P. R. China

**Keywords:** label‐free detection, silicon nanowires, single‐molecule devices, single nucleotide polymorphism

## Abstract

Establishing low‐cost, high‐throughput, simple, and accurate single nucleotide polymorphism (SNP) genotyping techniques is beneficial for understanding the intrinsic relationship between individual genetic variations and their biological functions on a genomic scale. Here, a straightforward and reliable single‐molecule approach is demonstrated for precise SNP authentication by directly measuring the fluctuations in electrical signals in an electronic circuit, which is fabricated from a high‐gain field‐effect silicon nanowire decorated with a single hairpin DNA, in the presence of different target DNAs. By simply comparing the proportion difference of a probe‐target duplex structure throughout the process, this study implements allele‐specific and accurate SNP detection. These results are supported by the statistical analyses of different dynamic parameters such as the mean lifetime and the unwinding probability of the duplex conformation. In comparison with conventional polymerase chain reaction and optical methods, this convenient and label‐free method is complementary to existing optical methods and also shows several advantages, such as simple operation and no requirement for fluorescent labeling, thus promising a futuristic route toward the next‐generation genotyping technique.

## 1 Introduction

Following decoding of the full sequence of human genomes in the Human Genome Project, investigating the variation among individual genomes and understanding the relationship between genetic variations and their biological functions on a genomic scale have attracted extensive attention from geneticists in the postgenomic era.^1^ Single nucleotide polymorphisms (SNPs) are prevalent and abundant genetic mutations. Because of their involvement in the emergence of numerous inherited diseases, SNPs have been used as genetic markers for mapping disease loci,^2^ and studying candidate gene association,^3^ revealing fundamental information for clinical diagnosis and drug discovery for related genetic diseases.^4^ Therefore, various genotyping techniques have been developed for SNP detection, such as allele‐specific real‐time polymerase chain reaction (PCR) assays,^5^ hybridization methods based on artificial DNA probes (molecular beacons, peptide nucleic acids (PNAs), and locked nucleic acids (LNAs)),^6^ enzyme‐assisted primer extension or chain ligation reaction genotyping by using DNA polymerase or ligase,^7^ and enzyme mismatch cleavages.^8^ However, most of these methods require pre‐ or post‐treatments such as complex and costly PCR or rolling circle amplification steps to generate large amounts of sample collection or for signal enhancement, significantly restricting their applications.^9^ To establish high‐throughout, simple, and accurate genotyping techniques, with the ultimate goal of detecting SNPs at the single‐molecule/single‐event level, it is necessary to develop next‐generation SNP‐genotyping technology based on single‐molecule analysis, which is promising for reducing the number of PCR amplification steps and lowering costs.^10^

Molecular beacons (MBs),[[qv: 6a,11]] which are derived from hairpin‐loop‐structured oligonucleotides containing a fluorophore and quencher at different ends of the strand, have been widely used for biological detection both in vitro and in vivo, such as polymorphism analysis, clinical diagnosis, genotyping, and allele discrimination.^12^ Because of their enhanced specificity and sensitivity, MBs are common and effective DNA probes for SNP genotyping. A single‐base mismatch can be easily distinguished by measuring the differences in fluorescence intensity induced by the mismatched site after binding to well‐matched and single‐base mismatched targets due to the dynamic diversities of MB hybridization with different targets. Comparing the thermal stability of probe targets and investigating an optimized temperature range to maximize signal differences can effectually improve the detection capability of MBs. However, different mismatched base sequences are difficult to identify,^13^ because in ensemble experiments involving equipments with limited sensitivity, the small differences in fluorescence intensity caused by tiny diversities in thermal stability may be averaged in the background noise, resulting in the failure to detect weak changes. To achieve allele‐specific discrimination, Kramer and co‐workers designed four different colored molecular beacons in multiplex hybridization experiments.^14^ However, fluorescence labeling is costly and complicated, hampering the large‐scale and low‐cost application of hairpin probes for SNP genotyping.

**Figure 1:**
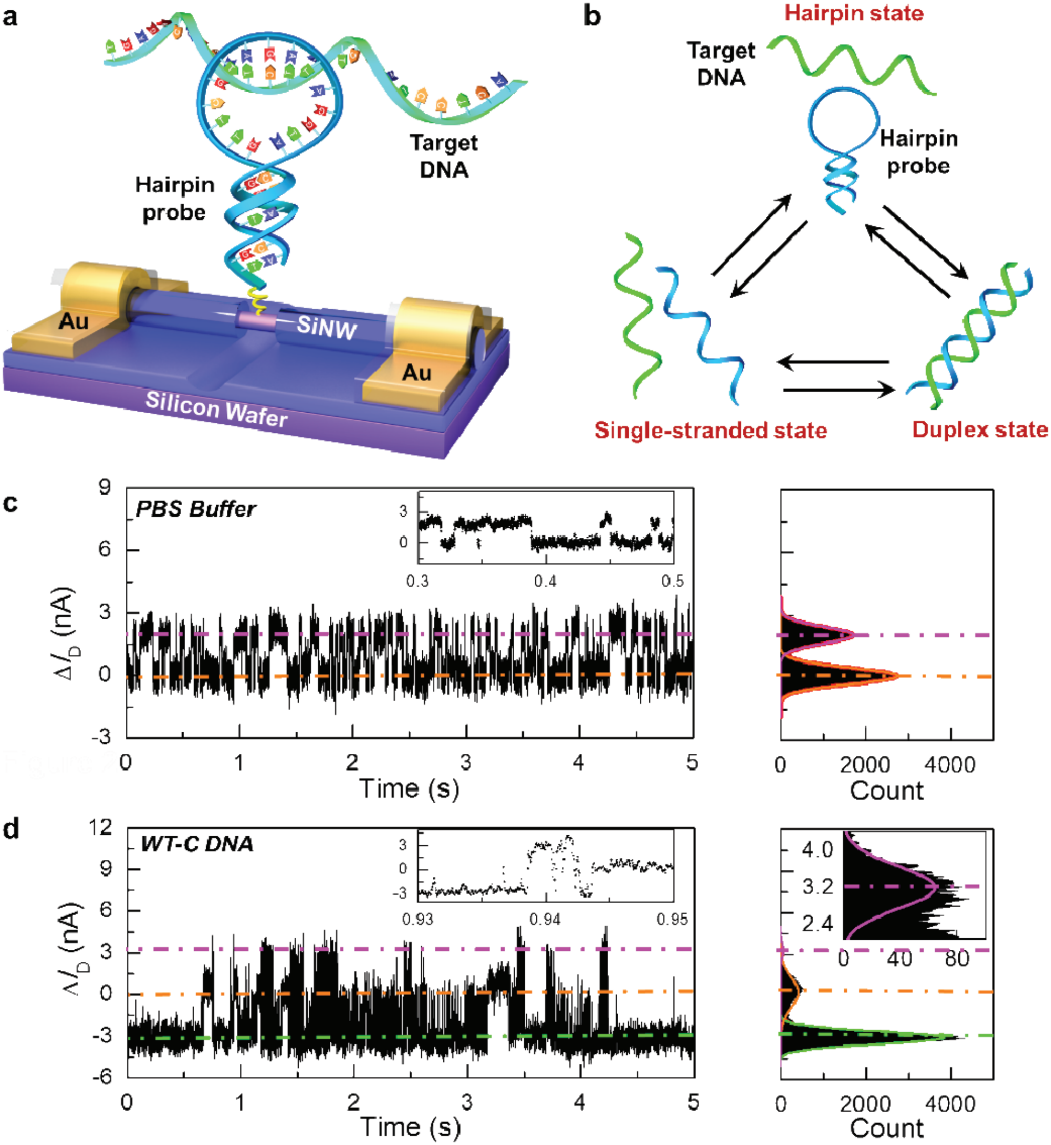
a) Schematic diagram of single‐molecule biosensors, where the interaction between the hairpin probe and the target DNA represents their hybridization process, not implying the conformation of the target binding to the hairpin loop. b) Schematic demonstration of three‐phase transitions during hairpin DNA hybridization with the complementary target. c,d) 5 s interval source–drain current fluctuations Δ*I*_D_(*t*) of a representative single hairpin DNA‐decorated SiNW biosensor measured in a pure PBS solution and a PBS solution containing 1 × 10^−6^ m complementary target (WT‐C DNA) at *T* = 45 °C, respectively. Insets show representative data over a short time interval. The right panels are the corresponding histograms of current values, revealing c) two and d) three Gaussian peaks in conductance.

In the past few years, single‐molecule detection based on electricity has emerged as a promising approach for single‐molecule dynamics studies.^15^ This approach is complementary to traditional optical methods but with the obvious advantages, such as no fluorescent labeling and no bleaching problem. In this direction, by incorporating a single hairpin DNA probe to a silicon nanowire (SiNW) field‐effect transistor (FET), we recently realized the dynamic monitoring of the folding/unfolding behavior of individual DNAs with single‐base pair resolution,[[qv: 15e]] demonstrating a robust single‐molecule platform with a high signal‐to‐noise ratio, high time resolution, and high bandwidth for studying biomolecular interactions. Considering the fact that hairpin‐loop‐structured DNAs are frequently used to design molecular beacons in optical gene detection as discussed above,[[qv: 6a]] in the current study we probed the detailed hybridization of individual hairpin DNAs with well‐matched and single‐base mismatched targets in a single‐molecule SiNW FET electronic circuit (**Figure** **1**a). We observed a three‐level fluctuation in device conductance at 45 °C, revealing the three‐phase transitions of hairpin hybridization with target DNAs. Next, by comparing the differences in dynamic parameters for probe‐target duplexes including the lifetime and proportion in the whole trajectory, SNP alleles were successfully discriminated. Our results prove that the dynamic differences between hairpin hybridizations with well‐matched and single‐base mismatched targets caused by very small diversities in the melting temperatures of duplex structures can be amplified by single‐molecule electrical measurements. In conjunction with other excellent features, such as the avoidance of both complicated PCR amplification steps and costly fluorescent labeling, the unique amplification ability demonstrates that our SNP genotyping electrical technology has the capability of the integration with current silicon industrial processing technology, suggesting the potential of fabricating large‐scale multiplexed microarrays in the future to achieve high‐throughput SNP genotyping in the whole genome.

## 2 Results and Discussion

SiNW‐based single‐molecule electrical biosensors (Figure 1a) were prepared by using a well‐developed strategy.[[qv: 15e,16]] The detailed process of device fabrication and DNA attachment is provided in the Experimental Section. Through well‐developed nanofabrication and biomolecular coupling strategies, we finally incorporated amino‐terminal hairpin DNAs on the sidewall of silicon nanowires. Here, SNPs of rs1007616 at chromosome 19q13.3, which are related to a lung cancer risk, were used as a test model.^17^ The sequences of hairpin and target DNAs were derived from the segments of rs1007616 (details are shown in Table S1, Supporting Information).

Hybridization assays with sequence‐specific oligonucleotide probes, such as molecular beacons, PNAs and LNAs, are common SNP genotyping techniques for identifying complementary and single‐base mismatched DNA strands. By probing the formation dynamics of probe‐target duplexes, the optimal stringency for discriminating SNP alleles can be achieved.^18^ In general, molecular beacons display three‐phase transitions (i.e., single‐stranded state, hairpin state, and probe‐target duplex state) upon interacting with target DNAs in solution (Figure 1b).^13^ Because temperature directly determines duplex structure stability in the hairpin stem and probe‐target complex, the temperature‐dependent profiles of fluorescence fluctuation caused by the concentration differences of the three conformations in solution provide a foundation for previous SNP detection. According to the melting curves of hairpin DNA (*T*_m_ ≈ 46.5 °C) and its hybrids with complementary target DNA (or wild‐type DNA, WT‐C, see Table S1, Supporting Information) (*T*_m_ ≈ 59.6 °C) and single‐base mismatched targets (mutant‐type DNAs with a mismatch site (A, G, or T), referred to as MT‐A, MT‐G, and MT‐T, respectively, Table S1, Supporting Information) we measured via UV–vis spectroscopy (Figure S2, Supporting Information), we used *T* = 45 °C as the measurement temperature to real‐time trace the conformational fluctuations of individual hairpin DNA hybridization with target DNAs.

All electrical measurements were performed on a home‐made temperature control apparatus (details are presented in the Experimental Section). After reaching the thermal equilibrium for ≈10 min, we applied a source–drain bias of 200 mV and zero gate bias, and then collected a long‐duration recording of time‐averaged currents. When the device was exposed to a pure phosphate‐buffered saline (PBS) solution, real‐time current recordings (Δ*I*_D_(*t*)) exhibited a large‐amplitude two‐level fluctuation (Figure 1c), where the conductance distribution could be fit into two Gaussian peaks. To compare the amplitude of the electrical fluctuations, we set the low‐conductance state as the baseline (“0”). As demonstrated in the previous report,[[qv: 15e]] the two‐level current oscillation in a PBS solution indicates the folding and unfolding processes of a hairpin DNA, wherein the high and low states in device conductance should be ascribed to the unfolded coil and hairpin state, respectively. This observation clearly demonstrates the success of point decoration of SiNWs by individual hairpin DNAs.

After proving the successful attachment of hairpin DNAs, the devices were thoroughly rinsed with PBS and then exposed to a PBS solution containing a complementary target DNA (1 × 10^−6^ m). It was found that the addition of target DNAs resulted in three‐level fluctuation behavior in time‐averaged current changes Δ*I*_D_(*t*) as shown in Figure 1d, where the conductance distribution could be fit into three Gaussian peaks. According to previous studies,[[qv: 15e,19]] the current oscillation was attributed to the alteration in either the scattering effect or charge transfer that were induced by duplex formation and dissociation at a single defect on naked silicon nanowires. Therefore, in combination with the two‐level fluctuation of hairpin DNAs, we propose a model for identification of the three‐level current oscillation: the low‐conductance (low) state represents the probe‐target duplex conformation, intermediate‐conductance (intermediate) state represents the hairpin conformation, and high‐conductance (high) state represents the single‐stranded conformation. The free energy diagram of hairpin hybridization also indicated that duplex conformation has the lowest free energy, followed by hairpin and single‐stranded conformations,^20^ implying the duplex state is the most stable conformation. On the other hand, the melting curve of the probe‐target duplex (*T*_m_ ≈ 59.6 °C) suggests that the unwinding probability of the duplex structure is relatively low at *T* = 45 °C (Figure S2, Supporting Information), resulting in fewer dissociation activities of the duplex conformation into the single‐stranded conformation or back to the hairpin conformation. Therefore, in the presence of well‐matched DNAs in solution, the hairpin DNA tends to form a duplex conformation.

**Figure 2:**
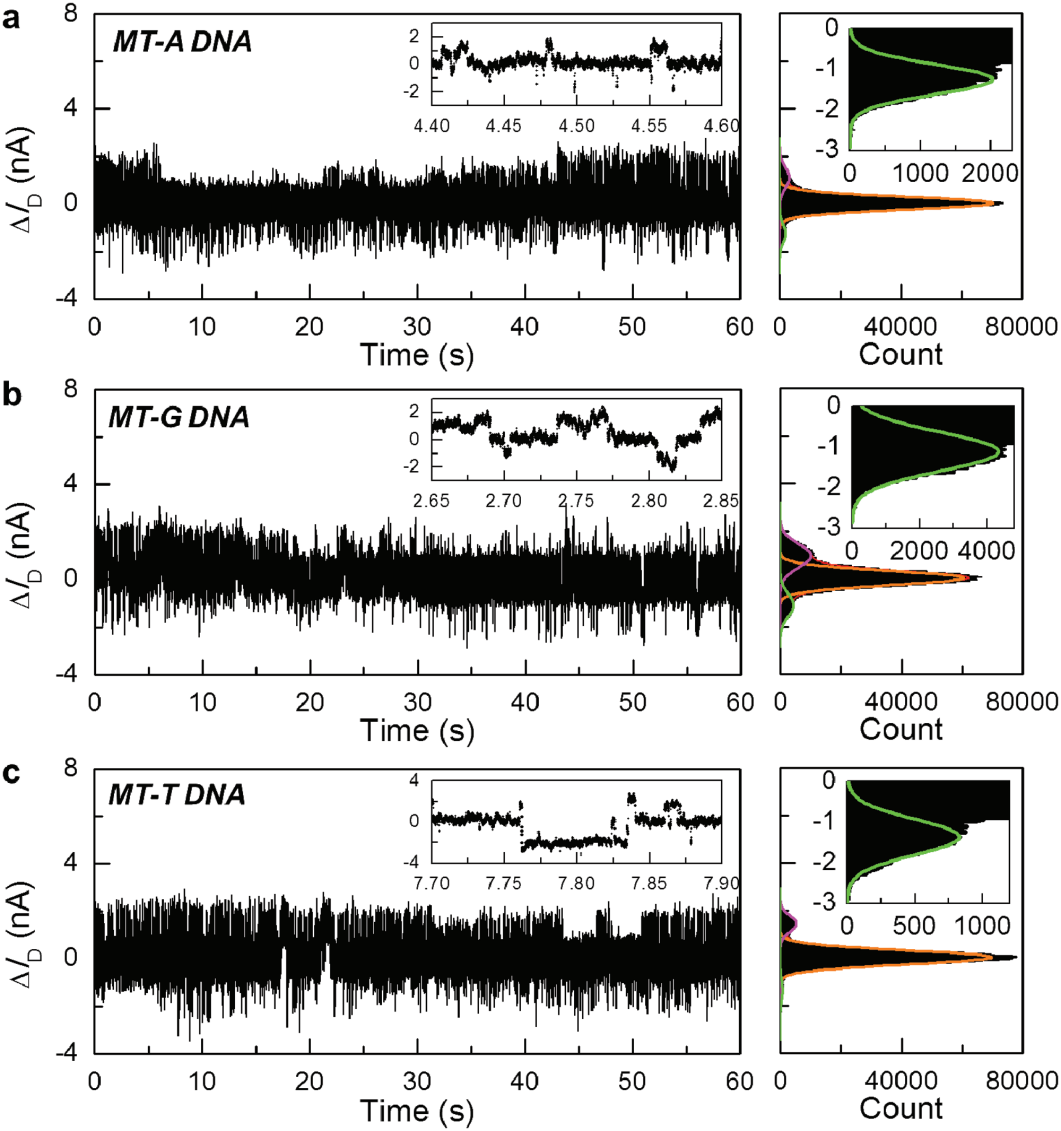
Hybridization for allele detection. Source–drain current fluctuations Δ*I*_D_(*t*) of the representative single hairpin DNA‐decorated SiNW biosensor in the presence of 1 × 10^−6^ m mismatched target DNA solution at *T* = 45 °C (a: MT‐A; b: MT‐G; c: MT‐T). The inserts in left panels are the amplified data over a short time interval. The right panels are the corresponding histograms of the conductance, revealing three Gaussian peaks. The inserts in right panels are the enlarged peaks for the low state.

In order to discriminate the SNP alleles, we carried out the electrical measurements by separately adding different single‐base mismatched target DNAs (MT‐A, MT‐G, and MT‐T, 1 × 10^−6^ m in the PBS solution) to the same device at *T* = 45 °C. As shown in **Figure** **2**a–c, all 60 s interval real‐time current fluctuations Δ*I*_D_(*t*) exhibited a distinct three‐level fluctuation, whose conductance distributions can be fit into three Gaussian peaks. In contrast with the case of well‐matched target DNAs, where the probe‐target duplex is the most stable, the intermediate state dominated the most, indicating that the hairpin state is the most stable in the presence of single‐base mismatched targets at *T* = 45 °C. This conformational difference is consistent with the fluorescence measurements based on molecular beacons.^21^ This distinct diversity should be ascribed to the cooperative effects of the weaker affinity of hairpin DNAs with single‐base mismatched targets and the less stable duplex structures formed by hairpin DNAs and mismatched targets. It is because of the weaker ability of opening the stem by mutant‐type targets that not every target binding attempt will completely undergo hairpin opening and duplex formation, resulting in a lower frequency of occurrence in the conformational transitions and simultaneously leaving more opportunities for hairpin folding and unfolding. In addition, the duplex structures formed between hairpin and single‐base mismatched targets are unstable due to the presence of a mismatched site and show greater unwinding probabilities. Therefore, they dissociate into the single‐stranded conformation or return back to the hairpin structure more easily. This leads to the smaller occupation proportion of the low state than that of the high state. We had observed similar three‐level fluctuating behaviors in ten different devices out of 13 independent measurements, indicating the reproducibility and reliability of the detection results. **Table** **1** lists the average values of three conductance states in five different groups of current recordings measured from the representative devices. Although the current amplitudes are not constant because of the different electrical performance of different SiNW FETs, it is important that the duplex conformation formed between the hairpin probe and mismatched target DNAs differs from that formed between the hairpin probe and well‐matched target DNAs because of the presence of a mismatch site, thus causing differences in current amplitudes in the same device.

**Table 1:** Averaging conductance and percentages of three states from five independent and representative electrical measurements based on different devices

Target DNA	Device state	No. 1		No. 2		No. 3		No. 4		No. 5		AVG P [%]
		ΔI [nA]	P [%]	ΔI [nA]	P [%]	ΔI [nA]	P [%]	ΔI [nA]	P [%]	ΔI [nA]	P [%]	
WT‐C DNA	Low	−3.09	76.2	−2.86	79.6	−2.56	81.3	−2.73	79.2	−2.95	76.4	78.5 ± 1.8
	Intermediate	0	21.7	0	19.3	0	17.9	0	18.9	0	23.1	20.2 ± 1.8
	High	3.19	2.1	2.91	1.1	2.85	0.8	3.08	1.9	3.23	0.5	1.3 ± 0.6
MT‐A DNA	Low	−1.35	1.2	−1.70	3.5	−2.29	4.2	−1.53	4.9	−2.09	4.2	3.6 ± 1.2
	Intermediate	0	90.4	0	90.0	0	89.6	0	88.8	0	90.8	90.0 ± 0.8
	High	1.36	8.4	2.21	6.5	1.46	6.2	1.68	6.3	1.79	5	6.4 ± 0.6
MT‐G DNA	Low	−1.21	6.3	−1.95	5.2	−2.10	9	−1.32	8.1	−1.98	11.7	8.3 ± 1.8
	Intermediate	0	70.6	0	78.5	0	72.5	0	71.4	0	68.5	72.0 ± 2.6
	High	1.33	23.1	2.12	16.3	1.52	18.5	1.75	20.5	2.08	19.8	19.7 ± 1.8
MT‐T DNA	Low	−1.38	1.6	−2.14	1.5	−2.37	1.4	−1.59	1.6	−2.23	1.7	1.6 ± 0.1
	Intermediate	0	92.7	0	90.6	0	88.4	0	90.0	0	90.8	90.5 ± 1.0
	High	1.25	5.7	2.40	7.9	1.56	10.2	1.39	8.4	1.89	7.5	7.9 ± 1.0

To confirm the specificity, we further carried out control measurements, where the device was exposed to a 1 × 10^−6^ m noncomplementary DNA solution (Non‐C DNA, which cannot bind to hairpin DNAs) (Table S1, Supporting Information). These measurements revealed only a two‐level fluctuation with two fit‐well Gaussian peaks in device conductance, which (we believe) results from the folding/unfolding process of hairpin DNAs (Figure S3, Supporting Information). Blank experiments, where we used a bare SiNW FET device that had been treated by hairpin DNAs after undecylenic acid immobilization but was not covalently functionalized with hairpin DNAs because of the lack of essential NHS‐esterification, demonstrated that when the blank device was exposed to PBS buffer, 1 × 10^−6^ m WT‐C, MT‐A, MT‐G, MT‐T, and Non‐C DNAs solutions, respectively, the current recordings Δ*I*_D_(*t*) exhibited no particular fluctuations with only a Gaussian distribution dominated by 1/*f* noise (Figure S4, Supporting Information). These results eliminate the possibilities of nonspecific surface absorption of either hairpin DNAs or ions in the electrolytic solution, thus support that the three‐level current oscillations originated from the intrinsic behaviors of hairpin DNA hybridization with the targets.

**Figure 3:**
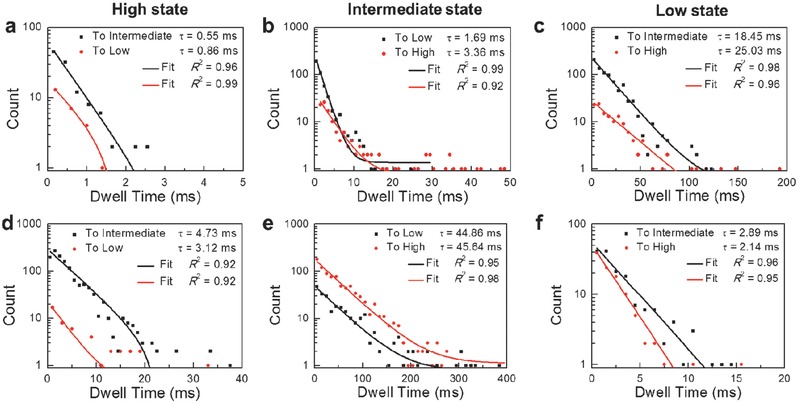
Distributions of the duration for the three conductance states: a–d) high state, b–e) intermediate state, and c–f) low state, extracted from the hybridization data of a hairpin DNA with WT‐C (above) and MT‐T (bottom), respectively. For each state, two distributions are shown to distinguish the direction of the conformational changes.

**Figure 4:**
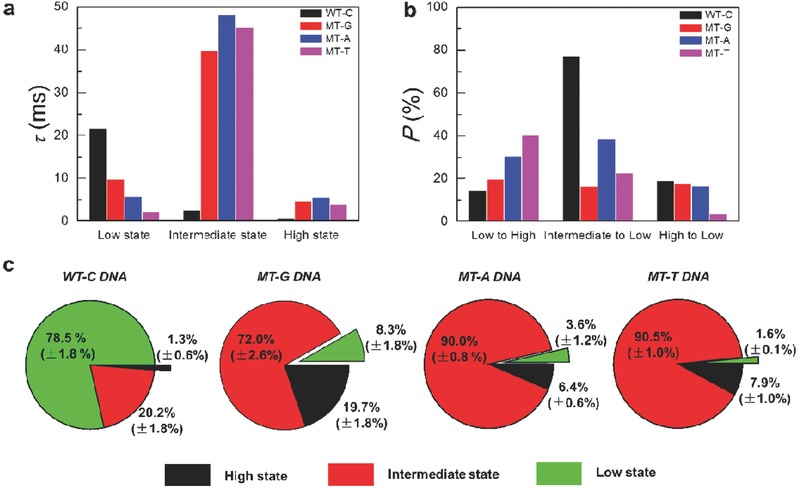
Comparisons of hybridization dynamics. a) Distributions of the mean lifetimes of each state. b) Distributions of the transition probability among the three states. c) Percentages of three conductance states of hairpin‐DNA hybridization in the presence of WT‐C, MT‐A, MT‐G, and MT‐T. Error bars were calculated from at least five groups of 5 s interval fluctuating data sets for a well‐matched target and 60 s interval for single‐base mismatched targets.

To achieve the discrimination of SNP alleles from a kinetic perspective, we idealized the three‐level fluctuation current data by using QUB software, which is a free software for Hidden Markov simulation and analysis of single‐molecule kinetics,^22^ and extracted the mean lifetimes (τ) of each state from the dwell‐time histograms. **Figure** **3**; and Figure S5 (Supporting Information) show the dwell‐time histograms and mean values for the duration of high, intermediate, and low states from the dynamic data of hairpin DNA hybridization with different targets. For a certain conformation showing two transition directions, the duration distribution can produce two lifetimes, depending on the following conformation. For instance, the duration of the high state followed by the intermediate state generates the folding rate from the single‐stranded conformation to the hairpin conformation, while that followed by the low state generates the hybridizing rate from the single‐stranded conformation to the duplex conformation. As demonstrated in Figure 3; and Figure S5 (Supporting Information), all six dwell‐time histograms extracted from the current data of hairpin DNA hybridization with well‐matched and single‐base mismatched targets can be fit to a single‐exponential decay function, respectively. **Table** **2** lists the mean lifetimes of each state and transition probabilities among three conformations. These results indicate the following conclusions: (1) The interaction between hairpin DNAs with well‐matched targets has the shorter average lifetimes of individual turnover than those with single‐base mismatched targets. This is because mismatched targets significantly slow down the hybridization dynamics due to their weaker affinity with hairpin DNAs. (2) Among the kinetic results of hairpin DNAs with well‐matched targets (WT‐C) and three mutant‐type targets (MT‐A, MT‐G, and MT‐T), the mean lifetimes of the low state display a sequence of τ_WT‐C_ > τ_MT‐G_ > τ_MT‐A_ > τ_MT‐T_ (**Figure** **4**a), indicating that the duplexes formed between hairpin and WT‐C DNAs are the most stable, successively followed by MT‐G, MT‐A, and MT‐T. The melting curves of probe‐mutant‐type target duplexes (Figure S2, Supporting Information) can explain these results. The probe‐WT‐C duplex has the highest melting temperature, so the lifetime is the longest, while the melting temperature of the probe‐MT‐T duplex is the lowest, allowing the target to more readily dissociate from the duplex. (3) The melting curves can also explain why most turnovers of the duplex conformation in the hybridization process of hairpin DNAs with MT‐T DNAs occurred during the transition to the single‐stranded conformation, followed by MT‐A, MT‐G, and WT‐C DNAs in sequence (Figure 4b). These comparisons suggest that the very small discrepancy in the melting behaviors of probe‐target duplexes at *T* = 45 °C can be amplified in single‐molecule electrical measurements of hairpin DNA hybridization dynamics, where the lifetimes and transition probabilities of the duplex conformation exhibit enhanced and regular distinctions, promising to be the basis for allele‐specific genotyping.

**Table 2:** Matrix of transition probabilities and mean lifetimes

Target DNA	Initial state	Final state					
		Low s tate		Intermediate state		High state	
		P [%]	τ [ms]	P [%]	τ [ms]	P [%]	τ [ms]
WT‐C DNA	Low			85.6	18.45 ± 1.36	14.4	25.03 ± 2.05
	Intermediate	77.4	1.69 ± 0.16			22.6	3.36 ± 0.57
	High	19.1	0.86 ± 0.14	80.9	0.55 ± 0.09		
MT‐A DNA	Low			69.7	8.97 ± 0.89	30.3	2.36 ± 0.60
	Intermediate	38.6	47.16 ± 5.17			61.4	49.16 ± 7.94
	High	16.6	3.00 ± 0.14	83.4	8.13 ± 0.56		
MT‐G DNA	Low			80.3	13.02 ± 2.01	19.7	5.66 ± 0.78
	Intermediate	16.4	32.93 ± 2.73			83.6	43.30 ± 3.57
	High	17.7	2.63 ± 0.13	82.3	6.58 ± 0.45		
MT‐T DNA	Low			59.7	2.89 ± 0.19	40.3	2.14 ± 0.09
	Intermediate	22.5	44.86 ± 3.43			77.5	45.64 ± 2.25
	High	3.5	3.12 ± 0.37	96.5	4.73 ± 0.46		

In addition to above‐discussed discrimination of SNP alleles from a kinetic perspective, we developed another direct and convenient comparative approach for distinguishing SNP alleles, based on the proportion differences of the three states among the hybridization process of hairpin DNAs with well‐matched and single‐base mismatched targets. We statistically extracted the percentages of each state from five groups of representative current recordings as listed in Table 1. As shown in Figure 4c, on average, the hairpin DNA spent most of the total time (78.5%) hybridizing with well‐matched targets, but a little time with single‐base mismatched targets in the order of MT‐G (8.3%) > MT‐A (3.6%) > MT‐T (1.6%). This is also evidenced by the diversity in the melting temperatures of probe‐target duplexes (*T*_m(WT‐C)_ > *T*_m(MT‐G)_ > *T*_m(MT‐A)_ > *T*_m(MT‐T)_, Figure S2, Supporting Information). Through directly comparing the differences in the proportion of the low state, we can visually discriminate SNP alleles, avoiding the use of multicolor fluorescence labeling.

Furthermore, we carried out electrical measurements to detect the number of the mismatched bases. Figure S6a,b (Supporting Information) showed a comparison of the current data of the hybridization dynamics between the hairpin DNA and a mutant‐type DNA with two mismatch sites (MT‐GT DNA) or three mismatch sites (MT‐GTG DNA) (Table S1, Supporting Information). Similarly, we calculated the percentage distributions of the three states from the 60 s interval data. As shown in Figure S6 (Supporting Information), the real‐time current data and corresponding percentage distributions indicated that as the number of mismatched bases increased the target's ability to open the stem and form the duplex conformation markedly decreased because of the decreasing affinity with the hairpin DNA, resulting in fewer hybridization events to form the duplex structure (low state) and distinct proportions of the high state in the total dynamic data. The control results in Figure S7 (Supporting Information) measured in the blank device eliminated the possibility of nonspecific surface absorption.

## 3 Conclusions

We have demonstrated a label‐free and convenient approach for SNP discrimination through directly measuring the dynamic electrical fluctuation of probe‐target duplex hybridization in a single hairpin DNA‐decorated SiNW FET electronic circuit. We accomplished the allele‐specific detection by simply comparing the proportion differences of the probe‐target duplex structure during the entire process, which can be strengthened by the dynamic differences including the mean lifetime and transition probability of the duplex conformation. These dynamics differences caused by the small diversity in the melting temperatures of duplexes can be amplified in single‐molecule hybridization measurements by an electrical approach in a nondestructive manner. This method avoids fluorescent labeling and perhaps reduces the PCR amplification steps, and holds the reliable capability of the integration with the semiconductor industry as well as in combination with machine learning and intelligent identification by fabricating automated and multiplexed microchips for high‐throughput and low‐cost SNP analysis, thus promising to be a futuristic candidate toward the next‐generation genotyping technique in the whole genome.

## 4 Experimental Section

*SiNW Growth and Device Fabrication*: The nanowire growth procedure is similar to those reported in the previous studies.[[qv: 15e,23]] Silicon wafers with a 300 nm thick thermal oxide layer were used as growth substrates and gold nanoparticles (AuNPs) with an average diameter of 20 nm (Ted Pella) were used as catalysts. Boron‐doped p‐type SiNWs were synthesized at 460 °C by using 2.5 sccm disilane (Matheson Gas Products, 99.998% Purity) as the reactant source, 0.13 sccm diborane (100 ppm, diluted in H_2_) as the p‐type dopant with a B/Si ratio of 1/100 000, and 7.5 sccm H_2_ as the carrier gas. High‐quality SiNWs were well‐aligned onto APTES ((3‐aminopropyl)triethoxysilane))‐assembled silicon wafer substrates with ≈1000 nm of thermally grown SiO_2_ on the surface by PDMS (polydimethylsiloxane)‐based microfluidic channels from the ethanol suspension. By using a standard lithography (BG‐401A, China electronics technology Group Corporation), the electrode patterns to individual SiNWs were opened. To form Ohmic electrical contacts with metal electrodes, a buffered HF solution (40% NH_4_F: 40% HF, 7:1) was used to remove the oxide shell of the nanowires. Then, metal leads (5 nm Cr followed by 60 nm Au) were formed through thermal evaporation (ZHD‐300, Beijing Technol Science). After metal deposition, another 50 nm thick SiO_2_ protective layer was deposited through electron beam thermal evaporation (TEMD‐600, Beijing Technol Science) before photoresist lift‐off for passivating the contact interfaces. In order to screen the drain current in the solution, it opened a negative resist (SU‐8, 2002) window by photolithography to protect the majority of the surface, exposing the SiNW region and contact pads. By using this method, it was able to make high‐density transistor arrays as shown in Figure S1a (Supporting Information). After SiNW transistors fabrication, electrical characterizations of these transistors were carried out at room temperature in the ambient by using an Agilent 4155C semiconductor analyzer and a Karl Süss (PM5) manual probe station. Figure S1b,c (Supporting Information) implies that SiNW transistors show the typical p‐type behaviors with good Ohmic contacts.

*Single‐Molecule Hairpin DNA Functionalization*: After SiNW FET fabrication, a PMMA layer (950, A4) was spincast (4000 rpm, 45 s) on the surface and then baked at 180 °C for 2 min. It used high‐resolution electron beam lithography to apply a designCAD file with a ≈5 nm wide line at the specific position to obtain the window precursor. The resist was developed in a mixture of water/isopropanol (V:V = 1:3) for the lift‐off at 4 °C for 1 min with the aid of sonication. After development, the devices were washed by deionized water and dried with a stream of N_2_ gas. Then, wet etching was processed by immersing the devices into the HF solution buffered with NH_4_F (40% NH_4_F: 40% HF, 7:1) for 10 s to completely remove the amorphous SiO_2_ layer in the window area, followed by a PMMA lift‐off by using acetone. Through sophisticated electron beam lithography and precise HF wet etching, a nanoscale window of hydrogen terminal on the side of SiNWs was obtained to confine a single biomolecule.

After gap opening, the devices were immediately annealed under an octadecyltrichlorosilane (OTS) vapor in the vacuum oven at 120 °C for 2 h to passivate reactive hydroxyl groups from the SiO_2_ surface and avoid the trap events from nonspecific absorption. After soaked in the n‐hexane solution overnight with the protection of an Ar gas, the devices were rinsed by ethanol and dried with a stream of N_2_ gas. Subsequently, the OTS‐treated devices and 3 mg powders of undecylenic acid were placed into a Schlenk bottle. After reaction under argon atmosphere at 90 °C for 10 h, the devices were soaked in dichloromethane and sonicated for 30 s to remove unreacted residues on the surface, and dried with a stream of N_2_ gas. After grafting, the devices were immersed in an aqueous mixed solution of *N*‐hydroxysuccinimide (NHS) (20 × 10^−3^ m) and 1‐ethyl‐3‐(3‐dimethylaminopropyl) carbodiimide (10 × 10^−3^ m) and allowed to react at room temperature for 1 h (pH = 6.5). Through hydrosilylation of Si‐H bonds with undecylenic acid and NHS‐esterification active ester terminals were obtained for the subsequent attachment of hairpin DNAs. The hairpin DNAs were purchased from Takara Biotechnology. The base sequence is H_2_N‐(CH_2_)_3_‐5′‐TGAGG ATGGA TAGAT GCTTG CCTCA‐3′, terminated with an amine group and a three‐carbon linker at the 5′ end. Through annealing at 90 °C in buffer, the five bases in both 5′ and 3′ ends can hybridize into a duplex and the single‐stranded DNA forms a hairpin configuration. Then, 50 µL hairpin DNA solution (1 × 10^−6^ m hairpin DNA in 10 × 10^−3^ m PBS (10 × 10^−3^ m Na_2_HPO_4_, 1.8 × 10^−3^ m KH_2_PO_4_, 2.7 × 10^−3^ m KCl, and 140 × 10^−3^ m NaCl, pH = 7.4)) was dropped on the surface of the devices for 12 h at 4 °C. Before used for electrical measurements, SiNW FETs were washed by the phosphate buffer solution (10 × 10^−3^ m, pH = 7.4) for 5 min.

*Electrical Measurement*: After rinsing single hairpin DNA‐decorated devices with the PBS solution, a PDMS chunk with a hole of ≈2 mm diameter was covered as a reaction chamber on the surface of the device and dropped a 50 µL solution into the chamber. The INSTEC hot/cold chuck with a proportion‐integration‐differentiation control system (±0.001 °C), which was recorded by a thermocouple in real time, and liquid nitrogen cooling system was used to maintain the testing temperature at *T* = 45 °C. The source–drain and gate biases were always held at 200 and 0 mV, respectively, by an HF2LI Lock‐in Amplifier (Zurich Instruments) in all the real‐time electrical measurements. The real‐time current was amplified by a DL1211 preamplifier operating at 10^8^ V/A gain and sampled by the HF2LI Lock‐in Amplifier equipped with a 5 kHz‐bandwidth low‐pass filter at a 28.8 KSa s^−1^ sampling rate. The DNA sequences used in this work are listed in Table S1 (Supporting Information).

*Melting Curve Measurement via UV–Vis*: The bulk melting curve for hairpin DNAs used here was measured by a UV–visible spectrophotometer (Lambda35, Perkin Elmer) with a circular heater/refrigerator. The temperature was monitored by a Fluke infrared thermometer. The sample and reference solutions in quartz cuvettes were a 1 × 10^−6^ m DNA sample solution in 10 × 10^−3^ m PBS solution (pH = 7.4) and a blank PBS solution, respectively. Prior to melting, the sample solution was first heated to 95 °C for 15 min and then placed on ice for 15 min. The testing temperature was maintained by the circular heater/refrigerator, varying from 15 to 80 °C, with a warming interval of 5 °C. After reaching the thermal equilibrium for 10 min, the ultraviolet absorption spectra were collected ranging from 180 to 300 nm at each temperature, with a peak in absorption intensity at 260 nm. The absorption was extracted at 260 nm as a function of temperature, and through normalization processing, the melting curves of the hairpin DNA and its hybrids with the targets were obtained, as shown in Figure S2 (Supporting Information).

## Conflict of Interest

The authors declare no conflict of interest.

## Supporting information

Supplementary

## References

[1] a) J. C. Venter , Science 2001, 291, 1304;11181995

[2] a) R. M. Bertina , B. P. C. Koeleman , T. Koster , F. R. Rosendaal , R. J. Dirven , H. de Ronde , P. A. van der Velden , P. H. Reitsma , Nature 1994, 369, 64; 8164741 10.1038/369064a0

[3] a) T. R. Rebbeck , M. Spitz , X. Wu , Nat. Rev. Genet. 2004, 5, 589; 15266341 10.1038/nrg1403

[4] a) L. J. Engle , C. L. Simpson , J. E. Landers , Oncogene 2006, 25, 1594; 16550159 10.1038/sj.onc.1209368

[5] M. V. Myakishev , Y. Khripin , S. Hu , D. H. Hamer , Genome Res. 2001, 11, 163. 11156625 10.1101/gr.157901 PMC311033

[6] a) S. Tyagi , F. R. Kramer , Nat. Biotechnol. 1996, 14, 303; 9630890 10.1038/nbt0396-303

[7] a) T. Pastinen , M. Raitio , K. Lindroos , P. Tainola , L. Peltonen , A.‐C. Syvänen , Genome Res. 2000, 10, 1031; 10899152 10.1101/gr.10.7.1031 PMC310927

[8] V. Lyamichev , A. L. Mast , J. G. Hall , J. R. Prudent , M. W. Kaiser , T. Takova , R. W. Kwiatkowski , T. J. Sander , M. de Arruda , D. A. Arco , B. P. Neri , M. A. D. Brow , Nat. Biotechnol. 1999, 17, 292. 10096299 10.1038/7044

[9] A.‐C. Syvanen , Nat. Genet. 2005, 37, S5. 15920530 10.1038/ng1558

[10] A.‐C. Syvanen , Nat. Rev. Genet. 2001, 2, 930. 11733746 10.1038/35103535

[11] W. Tan , K. Wang , T. J. Drake , Curr. Opin. Chem. Biol. 2004, 8, 547. 15450499 10.1016/j.cbpa.2004.08.010

[12] a) D. L. Sokol , X. Zhang , P. Lu , A. M. Gewirtz , Proc. Natl. Acad. Sci. USA 1998, 95, 11538; 9751701 10.1073/pnas.95.20.11538 PMC21676

[13] A. Tsourkas , M. A. Behlke , S. D. Rose , G. Bao , Nucleic Acids Res. 2003, 31, 1319. 12582252 10.1093/nar/gkg212 PMC150230

[14] S. Tyagi , D. P. Bratu , F. R. Kramer , Nat. Biotechnol. 1998, 16, 49. 9447593 10.1038/nbt0198-49

[15] a) S. Sorgenfrei , C.‐Y. Chiu , R. L. Gonzalez , Y.‐J. Yu , P. Kim , C. Nuckolls , K. L. Shepard , Nat. Nanotechnol. 2011, 6, 126; 21258331 10.1038/nnano.2010.275 PMC3783941

[16] J. Wang , F. Shen , Z. Wang , G. He , J. Qin , N. Cheng , M. Yao , L. Li , X. Guo , Angew. Chem., Int. Ed. 2014, 126, 5138. 10.1002/anie.201309438 24668898

[17] H. Ma , L. Xu , J. Yuan , M. Shao , Z. Hu , F. Wang , Y. Wang , W. Yuan , J. Qian , Y. Wang , P. Xun , H. Liu , W. Chen , L. Yang , G. Jin , X. Huo , F. Chen , Y. Y. Shugart , L. Jin , Q. Wei , T. Wu , H. Shen , W. Huang , D. Lu , Pharmacogenet. Genomics 2007, 17, 417. 17502833 10.1097/01.fpc.0000239975.77088.17

[18] a) A. V. Fotin , A. L. Drobyshev , D. Y. Proudnikov , A. N. Perov , A. D. Mirzabekov , Nucleic Acids Res. 1998, 26, 1515; 9490800 10.1093/nar/26.6.1515 PMC147416

[19] S. Sorgenfrei , C.‐Y. Chiu , M. Johnston , C. Nuckolls , K. L. Shepard , Nano Lett. 2011, 11, 3739. 21806018 10.1021/nl201781q PMC3735439

[20] G. Bonnet , A. Libchaber , Physica A 1999, 263, 68.

[21] G. Bonnet , S. Tyagi , A. Libchaber , F. R. Kramer , Proc. Natl. Acad. Sci. USA 1999, 96, 6171. 10339560 10.1073/pnas.96.11.6171 PMC26854

[22] C. Nicolai , F. Sachs , Biophys. Rev. Lett. 2013, 8, 191.

[23] F. Patolsky , G. Zheng , C. M. Lieber , Nat. Protoc. 2006, 1, 1711. 17487154 10.1038/nprot.2006.227

