# Supplementary material for: Single Nucleotide Polymorphism Genotyping in Single‐Molecule Electronic Circuits
Source: Adv Sci (Weinh). 2017 Jul 26;4(11):1700158. doi: 10.1002/advs.201700158 (PMC5700462; doi:10.1002/advs.201700158)
Supplement: Supplementary file 1 — Supplementary [file ADVS-4-na-s001.pdf]

Copyright WILEY-VCH Verlag GmbH & Co. KGaA, 69469 Weinheim, Germany, 2017.

ADVANCED SCIENCE
Open Access

Supporting Information

for *Adv. Sci.,* DOI: 10.1002/advs.201700158

Single Nucleotide Polymorphism Genotyping in Single-Molecule Electronic Circuits

*Gen He, Jie Li, Chuanmin Qi, and Xuefeng Guo**

Supporting Information

Single Nucleotide Polymorphism Genotyping in Single-Molecule Electronic Circuits

*By Gen He, Jie Li, Chuanmin Qi and Xuefeng Guo**

[*] G. He, J. Li, Prof. Dr. X. Guo
Beijing National Laboratory for Molecular Sciences, State Key Laboratory for Structural Chemistry of Unstable and Stable Species, College of Chemistry and Molecular Engineering, Peking University, Beijing 100871, P. R. China.
E-mail: guoxf@pku.edu.cn

G. He, J. Li, Prof. C. Qi
Key Laboratory of Radiopharmaceuticals, Ministry of Education, College of Chemistry, Beijing Normal University, Beijing 100875, P. R. China.

Prof. Dr. X. Guo
Department of Materials Science and Engineering, College of Engineering, Peking University, Beijing 100871, P. R. China.

Figures S1-7

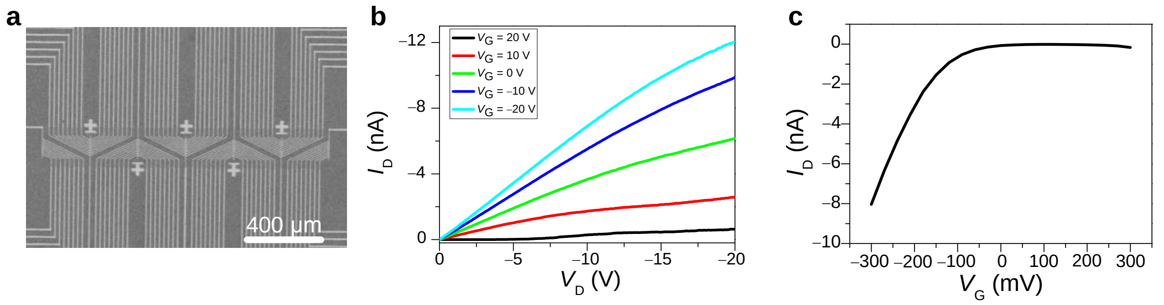

Figure S1. Device structure and electrical characterization. a) SEM image of a high-density SiNW-based transistor array. b) Output characteristics for a p-type SiNW FET device when V_D scans from 0 V to –20 V at different gate voltages (–20, –10, 0, 10 and 20 V). c) Current response of a single hairpin DNA-attached SiNW device to solution gating from 300 mV to –300 mV.

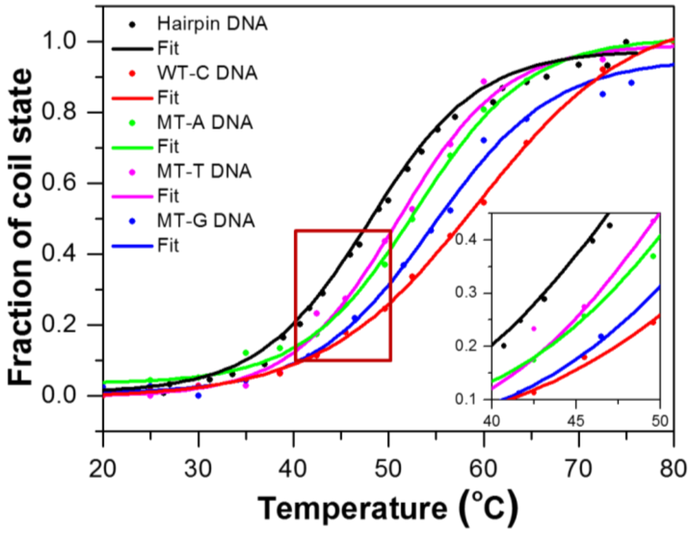

Figure S2. Melting curves of hairpin DNA and its hybrids with complementary and single-base mismatched target DNAs, implying that the melting behaviors of hairpin-DNA ($T_{m(hairpin)}$) and its hybrids with WT-C ($T_{m(WT-C)}$), MT-A ($T_{m(MT-A)}$), MT-G ($T_{m(MT-G)}$) and MT-T ($T_{m(MT-T)}$) are ~46.5 °C, ~59.6 °C, ~52.9 °C, ~55.4 °C and ~50.9 °C, respectively. Inset is the amplified figure of the red box zone.

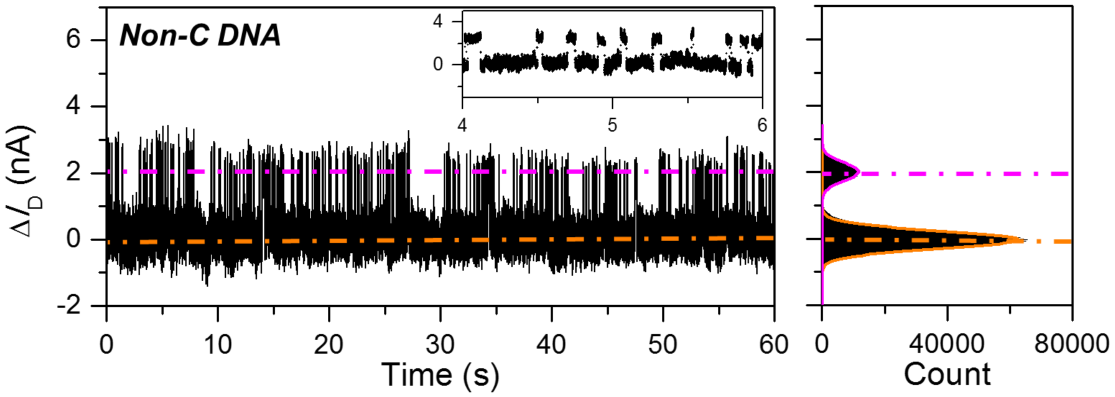

Figure S3. Control experiment using non-complementary DNA target (Non-C DNA). Source-drain current fluctuations $\Delta I_D(t)$ of the representative single hairpin DNA-decorated SiNW biosensor in the presence of a PBS solution containing 1 μM Non-C DNA (Table S1) at T = 45 °C. The insert in the left panel is the amplified 2s-interval data. The right panel is the corresponding histograms of the conductance for 60 s-interval data. In comparison with the experiment without target DNAs (Figure 1c), the experiment with non-complementary targets produced different populations. The reason might be that the structural fluctuation of the probe DNA could be affected by target DNAs to some extent due to the interactions between some complementary base pairs.

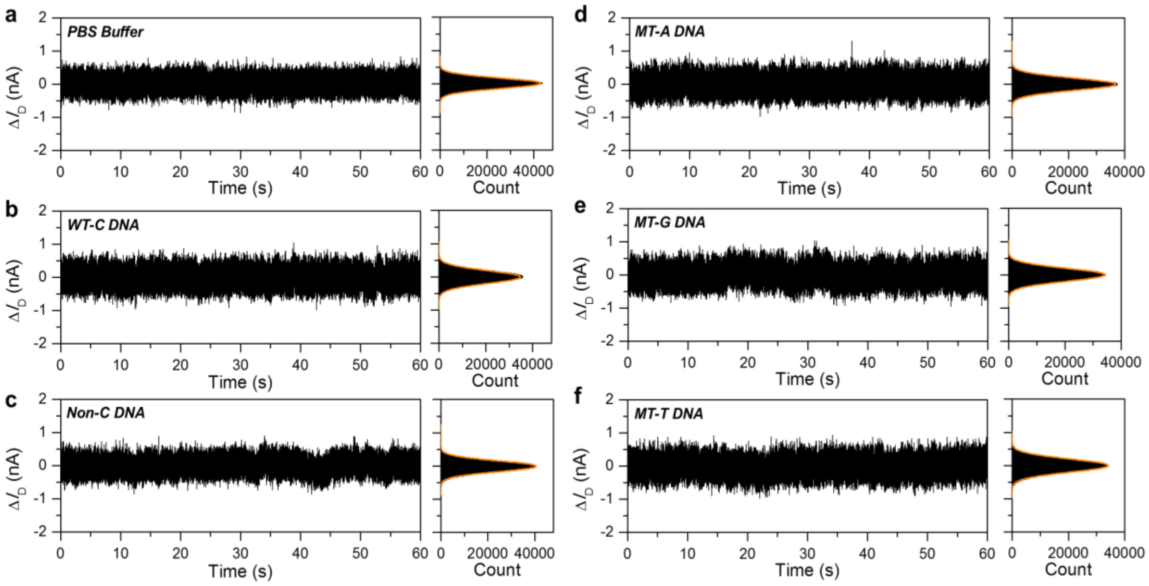

Figure S4. Control experiments. Source-drain current fluctuations ΔI_D(t) and corresponding histograms of the conductance in a hairpin DNA-free SiNW FET device in the presence of a pure PBS buffer solution (a), or a PBS solution containing 1 μM WT-C (b), Non-C (c), MT-A (d), MT-G (e) and MT-T (f) target DNAs at T = 45 °C, respectively. The left panels of each graph are real-time current fluctuations for a duration of 60 s. The right panels are the corresponding histograms of the conductance.

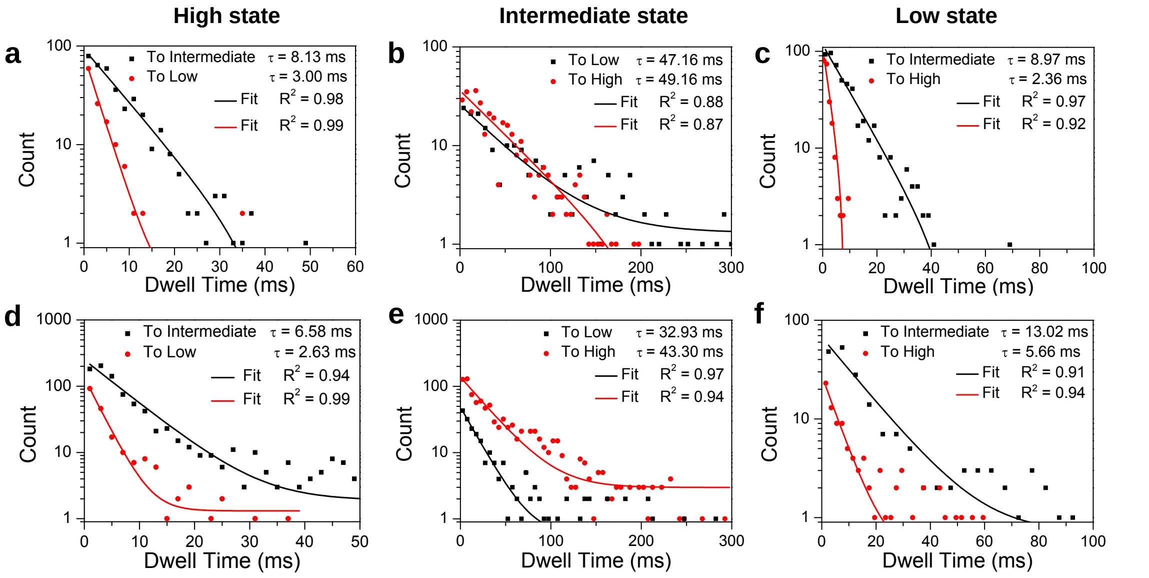

Figure S5. Distributions of the duration for the three conductance states: a-d) high state, b-e) intermediate state, and c-f) low state, extracted from the hybridization data of hairpin DNA with MT-A DNA (up) and MT-G DNA (bottom), respectively. For each state, two distributions are shown to distinguish the direction of the conformational changes.

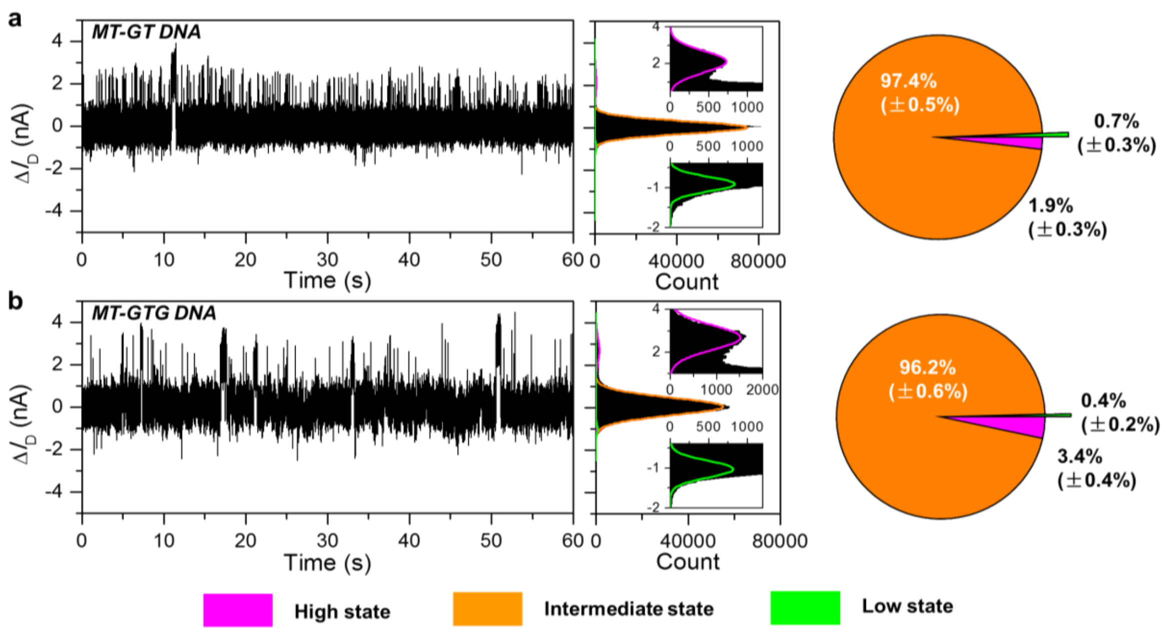

Figure S6. Detection of the mismatch number. Source-drain current fluctuations ΔI_D(t) and corresponding percentage distributions of the three conductance states of a representative single hairpin DNA-decorated SiNW biosensor in the presence of the PBS solution containing 1 μM MT-GT (a) and MT-GTG (b) target DNAs at T = 45 °C, respectively. The left panels of each graph are real-time current fluctuations for a duration of 60 s. The middle panels are the corresponding histograms of the conductance. Inserts are the amplified peaks for the low (green) and high (red) states. Error bars in the percentage distributions (right panels) were calculated from at least five groups of 60s-interval current recordings.

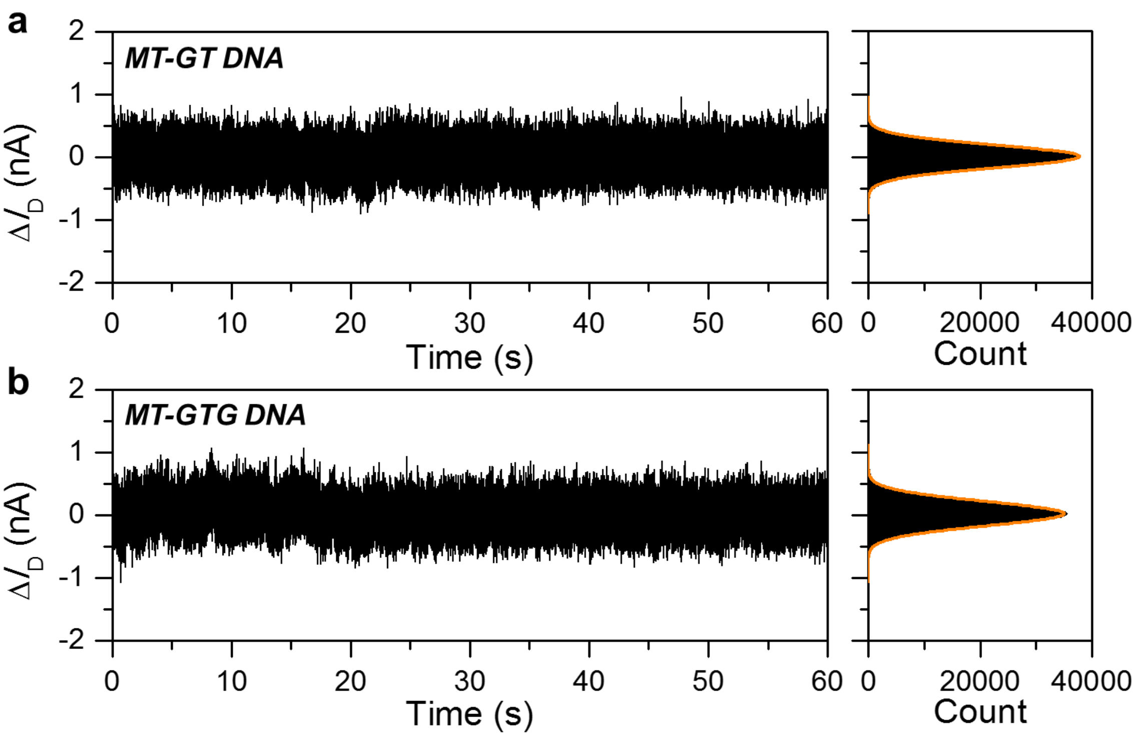

Figure S7. Source-drain current fluctuations ΔI_D(t) and corresponding histograms of the conductance in a hairpin DNA-free SiNW FET device in the presence of a PBS solution containing 1 μM MT-GT (a) and MT-GTG (b) target DNAs at T = 45 °C, respectively. The left panels of each graph are real-time current fluctuations for a duration of 60 s. The right panels are the corresponding histograms of the conductance.

Tables S1

Table S1. Sequences of hairpin DNA and target DNAs. The sequences with red marks are the mismatched bases.

DNA Name	Sequences (5'→3')
Hairpin Probe	TGAGG ATGGA TAGAT GCTTG CCTCA
Complementary DNA (WT-C)	TGAGG CAAGC ATCTA TCCAT CCTCA
Mutant-type DNA with a mismatch site A (MT-A)	TGAGG CAAGC ATATA TCCAT CCTCA
Mutant-type DNA with a mismatch site G (MT-G)	TGAGG CAAGC ATGTA TCCAT CCTCA
Mutant-type DNA with a mismatch site T (MT-T)	TGAGG CAAGC ATTTA TCCAT CCTCA
Mutant-type DNA with two mismatch sites GT (MT-GT)	TGAGG CAAGC AGTTA TCCAT CCTCA
Mutant-type DNA with three mismatch sites GTG (MT-GTG)	TGAGG CAAGC AGTGA TCCAT CCTCA
Non-complementary DNA (Non-C)	CAGTA GCCTC GCCGA GTAGT AAGTC
